# Duration of temporary catheter use for hemodialysis: an observational, prospective evaluation of renal units in Brazil

**DOI:** 10.1186/1471-2369-12-63

**Published:** 2011-11-17

**Authors:** Gisele MS Bonfante, Isabel C Gomes, Eli Iola G Andrade, Eleonora M Lima, Francisco A Acurcio, Mariângela L Cherchiglia

**Affiliations:** 1 Faculdade de Medicina, Universidade Federal de Minas Gerais, Brazil

## Abstract

**Background:**

For chronic hemodialysis, the ideal permanent vascular access is the arteriovenous fistula (AVF). Temporary catheters should be reserved for acute dialysis needs. The AVF is associated with lower infection rates, better clinical results, and a higher quality of life and survival when compared to temporary catheters. In Brazil, the proportion of patients with temporary catheters for more than 3 months from the beginning of therapy is used as an evaluation of the quality of renal units. The aim of this study is to evaluate factors associated with the time between the beginning of hemodialysis with temporary catheters and the placement of the first arteriovenous fistula in Brazil.

**Methods:**

This is an observational, prospective non-concurrent study using national administrative registries of all patients financed by the public health system who began renal replacement therapy (RRT) between 2000 and 2004 in Brazil. Incident patients were eligible who had hemodialysis for the first time. Patients were excluded who: had hemodialysis reportedly started after the date of death (inconsistent database); were younger than 18 years old; had HIV; had no record of the first dialysis unit; and were dialyzed in units with less than twenty patients. To evaluate individual and renal unit factors associated with the event of interest, the frailty model was used (N = 55,589).

**Results:**

Among the 23,824 patients (42.9%) who underwent fistula placement in the period of the study, 18.2% maintained the temporary catheter for more than three months until the fistula creation. The analysis identified five statistically significant factors associated with longer time until first fistula: higher age (Hazard-risk - HR 0.99, 95% CI 0.99-1.00); having hypertension and cardiovascular diseases (HR 0.94, 95% CI 0.9-0.98) as the cause of chronic renal disease; residing in capitals cities (HR 0.92, 95% CI 0.9-0.95) and certain regions in Brazil - South (HR 0.83, 95% CI 0.8-0.87), Midwest (HR 0.88, 95% CI 0.83-0.94), Northeast (HR 0.91, 95% CI 0.88-0.94), or North (HR 0.88, 95% CI 0.83-0.94) and the type of renal unit (public or private).

**Conclusion:**

Monitoring the provision of arteriovenous fistulas in renal units could improve the care given to patients with end stage renal disease.

## Background

Chronic hemodialysis requires permanent vascular access that can be used for months or years. The ideal permanent vascular access is the arteriovenous fistula (AVF). When AVF preparation is impossible, a graft can be used. Temporary vascular access can be obtained with cuffed or non-cuffed catheters. Catheters should be reserved for acute dialysis, when immediate access is needed, or for patients for whom permanent vascular access is problematic [1,2].

Continuing use of temporary catheters in patients on hemodialysis is the main concern of nephrologists in several countries; proper patient care requires that an AVF is functioning before renal replacement therapy (RRT) needs to begin [2,3]. Fistulas are associated with lower infection rates, lower hospitalization times and costs, better clinical results, a higher quality of life, and increased survival, compared to temporary catheters [4-8].

An AV fistula should be constructed at least 6 months prior to dialysis in order to permit maturation; a graft can be used after 3-6 weeks [2]. Regardless of the individual factors that influence the creation of a permanent vascular access, a temporary vascular access should be the last option for initial access and should be replaced by an AVF as soon as possible [2,3,8].

In Brazil, the proportion of patients with a temporary vascular access for more than 3 months is used a quality indicator for renal units, which are responsible for overseeing the placement of an AVF for their patients [9,10]. The National Kidney Foundation recommends that catheter use as the vascular access should be decreased to less than 10% of prevalent patients [2]. In Brazil in 2008, according to a study carried out by the Brazilian Nephrology Society, 11.4% of patients on hemodialysis had a catheter as the vascular access for hemodialysis [11]. No study in Brazil has tried to investigate the prolonged use of a temporary catheter among patients who started hemodialysis with this type of access.

Given the morbidity and mortality among patients with end-stage renal disease (ESRD) due to prolonged use of a temporary catheter, the aim of this study is to investigate the factors associated with the time between the beginning of hemodialytic treatment with a temporary catheter and the placement of the first AVF in Brazil between 2000 and 2004.

## Methods

### Data and Population Sources for the Study

This is an observational, prospective, non-concurrent study that used the National Database of RRT, which contains patient records for the 2000-2004 period [12,13]. This database was built using the subsystem data of the High Complexity/Cost Procedures Authority of the Outpatient Information System forms which are administrative records of all complex procedures financed by the Public Health System in Brazil. The forms are mandatory and filled out by the dialysis providers. This database includes 95% of all chronic dialysis patients in the country.

The total of incident patients in RRT from 01/01/2000 to 12/31/2004 who belonged to the National Database of RRT was 90,356. Of these, 72,155 started on hemodialysis. Two patients were excluded for whom hemodialysis reportedly started after the date of death. Four hundred and forty patients were excluded because of HIV, 1,874 were excluded because they were younger than 18 years old at baseline, 9,834 were excluded because they had no record of their first hemodialysis unit, and 4,416 because they were dialyzed in units with less than twenty patients. Ultimately, this study included 55,589 subjects.

Patients who were dialyzed in renal units where the number of patients was fewer than twenty were not included, because these units are probably not renal units, but hospitals that provide only emergency hemodialysis.

### Variables

The explanatory variables for each patient were sex, age, city (capital or interior) and region of residence in Brazil, cause of chronic renal disease (CKD) (glomerulonephritis, diabetes mellitus, hypertension and cardiovascular diseases, undetermined cause), and identification of his or her first renal unit. Variables related to the renal unit included: type (private vs. public), location (hospital-based vs. satellite) and whether or not the unit had rooms for outpatient surgeries.

As a response variable, the time, in months, between the beginning of hemodialytic treatment with a temporary catheter and the creation of the first AVF was used. In Brazil, the use of the arteriovenous vascular graft is not common, so the AVF is probably the choice for permanent vascular access.

### Statistical Analysis

A descriptive analysis was carried out, which included the distributions of the frequencies and measures of the central tendency. To check the association between the response variable and the explanatory variables, a separate curve of estimated survival was used for each explanatory variable, using the Kaplan-Meier method. Verification of the effect of each variable was evaluated using the Log-Rank test, which allowed for the selection of variables to build multiple models (p ≤ 0.05). Values for the median times and their 95% confidence intervals were obtained.

The Cox Regression Model was used to evaluate the individual factors associated with the time until the first AVF procedure. In addition, differences among the renal units represent an important factor in the quality of the treatment provided to the patients. Therefore, considering that patients treated in the same unit would be exposed to a common risk of occurrence for the event of interest, the frailty model was applied. By means of the inclusion of a random effect (frailty) at the renal unit level, this model allowed for the insertion of explanatory variables related to these services that estimate the patients' risk for a given unit, in addition to correcting for the effects of the other variables. The power of association for survival models was demonstrated by the hazard risk and the models' respective 95% confidence intervals [14].

We used the significance value of the unit variables and the likelihood ratio test to choose which unit variable would be the frailty variable. Thus, the survival model without frailty was tested with each unit variable separately.

To compare the obtained survival models with and without frailty, in addition to the theoretical hypothesis that the renal services play a key role in providing quality care to these patients, the likelihood ratio test was also used. The software R 2.12.1 was used for the statistical analysis.

This investigation is part of the RRT Project, "Economic-Epidemiologic Evaluation of the Renal Replacement Therapy Modalities in Brazil," developed by the Health Economics Research Group of the Federal University of Minas Gerais, approved by the Ethics in Research Committee of the Federal University of Minas Gerais.

## Results

The patients (N = 55,589) were distributed in 606 renal units. The average age of the patients was 53.95 ± 16.01 years. Approximately 57% of these patients maintained a temporary catheter until the censored date (end of the study or date of dead or date of transplant). Among the 23,824 (42.9%) patients who underwent fistula placement in the period of the study, 18.2% maintained the temporary catheter for more than three months until the fistula creation. The average time until the placement of the first AVF for these patients (N = 23,824) was 3 ± 6.26 months. Other characteristics are presented in Table 1.

**Table 1:** Characteristics of incident hemodialysis patients and renal units, National RRT Database, Brazil, 2000-2004.

Variables	N	N % (Valid)
**Individuals**	**55,589**	**100.0**
***Socio-demographics***		
*Sex*		
Male	31,925	57.4
Female	23,664	42.6
*Year of entry*		
2000	10,957	19.7
2001	11,876	21.4
2002	12,452	22.4
2003	12,579	22.6
2004	7,725	13.9
*Region of residence*		
Southeast	27,32	49.1
Northeast	13,779	24.8
South	8,844	15.9
Northern	2,701	4.9
Midwest	2,945	5,3
*City of residence*		
Interior	35,579	64.0
Capital	18,312	32.9
*Missing*	1,698	3.1
***Clinics ***		
*Cause of Chronic Kidney Disease*		
Undetermined	23,336	42.0
Hypertension and Cardiovascular Diseases	13,824	24.9
Diabetes Mellitus	8,825	15.9
Glomerulonephritis	6,901	12.4
Others	2,703	4.9
**Renal units**	**606**	**100**
*Type*		
Private	507	83.7
Public	99	16.3
*Location*		
Hospital-based	247	40.8
Satellite unit	359	59.2
*Existence of rooms for outpatient surgeries*		
Yes	186	30.7
No	420	69.3

Source: National RRT Database

In the univariate analysis, it was observed that 4 variables at the individual level and 3 variables at the renal unit level were significantly associated with the time to the first AVF (Table 2).

**Table 2:** Association between time to first fistula placement and explanatory variables, Brazil, 2000-2004†.

Explanatory variables	Median	95% CI	p-value*
***Level 1: Individuals***			
*Sex*			0.51
Male	30.2 ± 0.2	29.8-30.5	
Female	29.4 ± 0.2	29-29.8	
*Cause of Chronic Kidney Disease*			0.000*
Glomerulonephritis	1.73 ± 0.005	1.72-1.74	
Diabetes Mellitus	1.76 ± 0.004	1.76-1.77	
Hypertension and Cardiovascular Diseases	1.75 ± 0.004	1.74-1.75	
Undetermined and others	1.76 ± 0.003	1.76-1.77	
*City of residence*			0.000*
Interior	1.75 ± 0.002	1.75-1.75	
Capital	1.76 ± 0.003	1.75-1.76	
*Region of residence*			0.000*
South	1.78 ± 0.004	1.78-1.79	
Northeast	1.76 ± 0.004	1.75-1.77	
North	1.77 ± 0.008	1.76-1.79	
Southeast	1.74 ± 0.003	1.74-1.75	
Midwest	1.77 ± 0.008	1.75-1.78	
***Level 2: Dialysis units***			
*Type of provider*			0.000*
Private	1.75 ± 0.002	1.75-1.75	
Public	1.78 ± 0.005	1.77-1.79	
Location			0.000*
Hospital-based	1.78 ± 0.003	1.77-1.78	
Satellite	1.74 ± 0.002	1.74-1.75	
*Existence of rooms for outpatient surgeries*			0.000*
Yes	1.77 ± 0.003	1.77-1.78	
No	1.75 ± 0.002	1.74-1.75	

† To survival analysis was used Kaplan-meyer

* p ≤ 0.05 (Log-rank test)

Source: National RRT Database

Table 3 shows the Cox models with and without frailty. The value of the likelihood ratio test for the unit variables tested in frailty models was 546 for the "type of renal unit", 529 for the "location" of these units and 459 for the "existence of rooms for outpatient surgeries". Thus, the unit-level variable that proved to be most associated with the time until the placement of the first AVF was the type of renal unit. The inclusion of frailty in the model proved to be statistically significant and led to an increase in the Likelihood Ratio Test statistic from 398.8 to 546, showing the importance of evaluating the influence of the renal units upon providing a permanent vascular access and thus justifying the choice of the frailty model.

**Table 3:** Cox models for time to first fistula placement, Brazil, 2000-2004.

	No Frailty			Frailty		
Explanatory variables	Hazard-risk	95% CI	p-value	Hazard-risk	95% CI	p-value
***Individuals***						
*Age*	0.99	0.99-1.00	*	0.99	0.99-1.00	*
*Cause of Chronic Renal Disease *†						
Diabetes Mellitus	1.02	0.98-1.07	0.34	1.01	0.96-1.06	0.68
Hypertension and Cardiovascular Diseases	0.94	0.90-0.99	*	0.94	0.90-0.98	*
Undetermined and others	0.91	0.88-0.95	*	0.91	0.87-0.95	*
*City of residence *†						
Interior	0.91	0.89-0.94	*	0.92	0.90-0.95	*
*Region of residence*†						
South	0.85	0.82-0.88	*	0.83	0.80-0.87	*
Midwest	0.88	0.82-0.93	*	0.88	0.83-0.94	*
North	0.89	0.83-0.95	*	0.88	0.83-0.94	*
Northeast	0.91	0.89-0.94	*	0.91	0.88-0.94	*
***Renal units***						
*Type of renal unit (frailty variable)*						
Likelihood Ratio Test	398.8			546		
Frailty variance	-			0.55		

Source: National RRT Database

† Reference categories respectively: glomerulonephritis, capital and Southeast.

*p ≤ 0.05

Therefore, in the final model, the following variables remained: age, cause of CKD, city, region of residence, and type of renal unit.

It was observed that older patients tend to have an approximately 1% lower probability for the placement of the first AVF for each additional year. Patients with hypertension and cardiovascular diseases as a cause of CKD had a lower probability of permanent vascular access by approximately 6% compared to patients with glomerulonephritis.

Patients who lived in capital cities had an 8% lower probability for the creation of the first AVF compared to those who lived in the interior cities. Residents of the South, Midwest, Northern, Northeast regions also had a reduced probability of approximately 17%, 12%, 12% and 9%, respectively, in relation to the residents of the Southeast.

**Figure 1:**
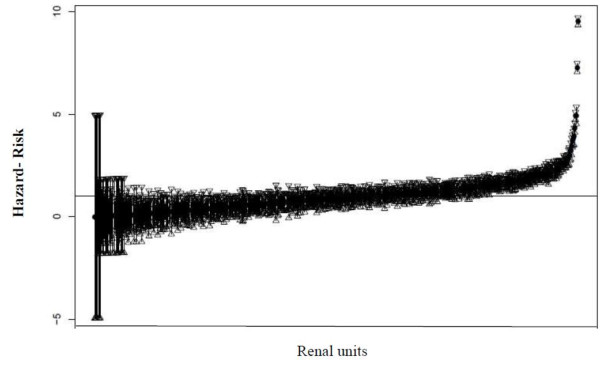
**"Risks of renal units and their 95% CI to first fistula placement, Brazil, 2000-2004"**. This figure shows an estimative of frailty risks of renal units and their respective 95% confidence intervals of the time to completion of the first fistula, Brazil, 2000-2004.

It was observed that the frailty variance was significant, indicating variability in the probability of occurrence of the event among renal units (0.55). Figure 1 shows the frailty estimates for the hazard risks for each of the 606 renal units, which can be classified into 3 groups: frailties < 1, middle frailties, and frailties > 1.

Patients whose renal units had a frailty < 1 tended to have longer waits for AVF surgery, whereas a frailty > 1 increased the probability of having surgery [14].

## Discussion

In the present study, the proportion of patients on hemodialysis who continued to use the catheter for a period > 3 months was still above the recommended level, as reported in Sesso et al. (2008) [2,11]. Several countries have made efforts to reduce this proportion, such as the United States, where one third of patients are still on dialysis with a catheter after 6 months from the beginning of treatment [6,8,15]. Among European countries, differences in catheter use can also be observed, though some are close to, or have already reached, the established practice standards [8].

The average time until AVF placement in our study was greater than that found in the study by Linardi et al. (2004), whose estimate varied from 1 to 3 weeks for Brazil [16]. The study by Either et al. (2008) reported that the minimum time was 5 days for Italy and 43 days for the United Kingdom [8].

The results of this analysis indicated 4 individual factors that were associated with the time until the first AVF procedure: age; having hypertension and cardiovascular diseases or undetermined cause as causes of CKD; residing in capital cities; and residence in certain regions of Brazil (South, Midwest, North, and Northeast regions). These associations remained significant even after the inclusion of the variable related to renal unit (type of renal unit).

Increasing age was associated with longer times with temporary catheters, possibly because older patients were more likely to have vascular disease, making fistulas more difficult to establish, as suggested by several authors [4,15,17]. Reddan et al. (2002) suggest that AVF failure rates may be increased in older patients [17]. On the other hand, Stehman-Brenn et al. (2000) point out that older age does not necessarily result in poorer patency outcomes [15].

Hypertension was associated with a lower probability for the AVF procedure in relation to glomerulonephritis, in agreement with other studies that address hypertension [18]. This proposition is plausible for Brazil, where hypertensive patients face difficulties in obtaining pre-dialysis care, which reduces their chances of having a mature access at the time of dialysis [19].

The present study also found that patients who lived in capital cities had a lower probability of having timely performance of fistula surgery. A study by Osis et al. (1993) found a higher use of services by the inhabitants of the interior cities (medical appointments) which may be associated with differences in the access to care [20]. Therefore, for our study, residing in interior cities could result in less competition for the performance of the outpatient procedure. However, another study diverges from this finding and reports easier access for the inhabitants of the capitals, indicating that these cities may have a higher technological density and capacity [21].

Brazil is divided into five regions: Southeast, South, Midwest, Northern and Northeast. This division emphasizes a historical and spatial perspective, referring to economic and social characteristics and political organization of the national space [22]. The differences found among the regions of Brazil point to differences in access to health care in the country. Coelho et al. (2006) point to a large discrepancy in the health indicators among the South and Southeast regions, which present better results, and the Northern and Northeast regions, which may suggest differences in access to health services [21]. The analysis carried out by Cazelli et al. (2002) also revealed differences among the regions in the availability of high complexity equipment, where the Northern and Northeast regions showed lower coverage than the national average [23].

Lima et al. (2002) suggests that one of the factors related to the differences found in the access to and use of health services among the regions may be the differences in the size and complexity of the service networks, which influence their availability [24].

Studies conducted in England and the United States also found a wide variability in the use of the AVF among different geographic regions of the countries, suggesting that this variation reflects differences in the practice standards adopted by nephrologists and vascular surgeons or renal unit teams; it may also suggest discrepancies between the offer and demand for renal units and the variations in the type or quality of the services offered [4,25,26].

The variations observed in the time until placement of AVF were associated with the type of renal units. This allows for the identification of an association between the renal service and the provision of the fistula, which can be related to differences in the practice standards of these services [14]. The variation found among the renal units confirms the importance of including evaluations of these services to identify points of reference for public policies that can impact the quality of health care for ESRD patients.

Linardi et al. (2003) found variations in catheter use among renal units that were distributed among 7 Brazilian states and suspected that structural differences among units, such as the location of the clinic in relation to the regional reference hospital, could influence the patients' profiles [27].

A study by Allon et al. (2000) also showed differences in the prevalence of fistulas among different renal units in a metropolitan area of the United States. However, the data collected in this study did not allow an evaluation the reasons of this variability, and stated that specific renal units have been able to increase the fistula frequency through the implementation of their own efforts [4].

Limitations inherent in the use of an administrative database should be considered for the present investigation because factors such as socioeconomic level, race or ethnicity, and co-morbid conditions could not be investigated. In addition, we used the dependent variable of the date of fistula surgery, and not the date of catheter removal, because in the National Database of RRT there are only the dates of the creation of the first AVF. However, we must consider that the date of catheter removal would be more clinically relevant, because even a successful fistula will not go into service for 6-8 weeks and many fistulas do not mature. Thus, the proportion of those with the first AVF surgery included some patients who have had their first surgeries but for whom the maturation of the AVF "failed".

The patients who had an undetermined cause for CKD were not excluded from the analysis because, as suggested by Moura et al. (2009), the exclusion of patients with this condition (42%) could compromise the present investigation [28]. Moreover, this study showed a deficiency in this subsystem of the Brazilian Public Health System for recording the causes of CKD. The proportion found in this study and in Sesso et al. (2008) was practically double the proportion reported in Spain and is higher than the proportion in the United States [11,15,18,29].

## Conclusion

The differences in the probability of time until the first fistula placement detected in this investigation were associated with individual characteristics and with the renal units where hemodialysis was performed. These associations reinforce the need to include monitoring of the provision of permanent vascular access in renal units to evaluate the quality of these services (it is an essential quality indicator of health care), with the objective of obtaining better results in the care provided by the Brazilian Public Health System to patients with CKD. Future investigations that further evaluate these services are of great importance for the improvement of patient care.

## List of abbreviations

AVF: arteriovenous fistula; RRT: renal replacement therapy; ESRD: end-stage renal disease; HIV: human immunodeficiency virus; CKD: chronic kidney disease.

## Competing interests

The authors declare that they have no competing interests.

## Authors' contributions

First, all authors read and approved the final manuscript. **GMSB**: Conception, design, analysis and interpretation of data. Drafting the article. Providing intellectual content of critical importance to the work described. **ICG**: Data collection and analysis of data. Providing intellectual content of critical importance to the work described. **EIGA**: Analysis and interpretation of data. Revision of the article. Providing intellectual content of critical importance to the work described. **EML**: Analysis and interpretation of data. Revising the article for critically important intellectual content. Providing intellectual content of critical importance to the work described. **FAA**: Analysis and interpretation of data. Revision of the article. Providing intellectual content of critical importance to the work described. **MLC**: Conception, design, analysis and interpretation of data. Drafting the article. Providing intellectual content of critical importance to the work described.

## Authors' information

**GMSB: **Masters in Public Health

e-mail: gimacedosilva@.hotmail.com

**ICG: **Masters in Statistics

e-mail: bebel_suty@yahoo.com.br

**EIGA: **Ph.D in Demography

e-mail: iola@medicina.ufmg.br

**EML: **Ph.D in Medicine (Nephrology)

e-mail: eleonoralima@uol.com.br

**FAA: **Ph.D in Animal Science

e-mail: acurcio@medicina.ufmg.br

**MLC: **Ph. D in Public Health

e-mail: cherchml@medicina.ufmg.br

Affiliation of all authors: Faculdade de Medicina, Universidade Federal de Minas Gerais

## Pre-publication history

The pre-publication history for this paper can be accessed here:

http://www.biomedcentral.com/1471-2369/12/63/prepub
